# High Temperature—Short Time Pasteurization Has a Lower Impact on the Antiviral Properties of Human Milk Than Holder Pasteurization

**DOI:** 10.3389/fped.2018.00304

**Published:** 2018-10-16

**Authors:** Manuela Donalisio, Massimo Rittà, Rachele Francese, Andrea Civra, Paola Tonetto, Alessandra Coscia, Marzia Giribaldi, Laura Cavallarin, Guido E. Moro, Enrico Bertino, David Lembo

**Affiliations:** ^1^Laboratory of Molecular Virology, Department of Clinical and Biological Sciences, University of Turin, Turin, Italy; ^2^Neonatal Intensive Care Unit, Department of Public Health and Pediatrics, University of Turin, Turin, Italy; ^3^Consiglio Nazionale delle Ricerche-Istituto di Scienze delle Produzioni Alimentari, Bari, Italy; ^4^Consiglio per la Ricerca in Agricoltura e l'Analisi dell'Economia Agraria, Centro di Ricerca in Ingegneria e Trasformazioni Agroalimentari, Turin, Italy; ^5^Italian Association of Human Milk Banks, Milan, Italy

**Keywords:** human milk, HTST, Holder, antiviral activity, virus, pasteurization

## Abstract

Holder pasteurization (62. 5°C for 30 min) is recommended by all international human milk bank guidelines to prevent infections potentially transmitted by donor human milk. A drawback is that it affects some human milk bioactive and nutritive components. Recently, High Temperature-Short Time (HTST) pasteurization has been reported to be a valuable alternative technology to increase the retention of some biological features of human milk. Nevertheless, to date, few data are available about the impact of pasteurization methods other than Holder on the antiviral activity of human milk. The present study was aimed at evaluating the antiviral activity of human milk against a panel of viral pathogens common in newborns and children (i.e., herpes simplex virus 1 and 2, cytomegalovirus, respiratory syncytial virus, rotavirus, and rhinovirus), and at assessing the effect of Holder and HTST pasteurization on milk's antiviral properties. The results indicate that human milk is endowed with antiviral activity against all viruses tested, although to a different extent. Unlike the Holder pasteurization, HTST preserved the inhibitory activity against cytomegalovirus, respiratory syncytial virus, rotavirus and herpes simplex virus type 2. By contrast, both methods reduced significantly the antiviral activities against rhinovirus and herpes simplex virus type 1. Unexpectedly, Holder pasteurization improved milk's anti-rotavirus activity. In conclusion, this study contributes to the definition of the pasteurization method that allows the best compromise between microbiological safety and biological quality of the donor human milk: HTST pasteurization preserved milk antiviral activity better than Holder.

## Introduction

A mother's own milk is the first choice for improving the short- and long-term outcomes for all newborns, including preterm infants (1, 2). When a mother's own milk is unavailable or in short supply, a common occurrence in Neonatal Intensive Care Units, the World Health Organization and the American Academy of Pediatrics recommend the use of donor milk (DM) as the best alternative (2, 3). Human milk (HM) can be considered a species-specific dynamic biological system, known to encompass many kinds of biological functions, including antimicrobial and antiviral properties (2). It is generally agreed that breastfeeding reduces the rate of serious gastroenteritis, especially caused by rotaviruses (HRoVs), and infant respiratory infections, as well as otitis media. The main viruses involved in infant respiratory and middle ear infections are respiratory syncytial virus (RSV) and rhinoviruses (HRhV) (4–7). Furthermore, most herpetic infections are acquired during childhood and their infection is lifelong. The vast majority of herpes simplex virus 1 (HSV-1) infections are oral-labial herpes and they are mainly transmitted by oral-to-oral contact. By contrast, neonatal herpes can occur when an infant is exposed to herpes simplex virus 2 (HSV-2) in the genital tract during delivery. The risk for neonatal herpes is greatest when a mother acquires HSV infection for the first time in late pregnancy (8). Human cytomegalovirus (HCMV) is another herpesvirus, responsible for the most common congenital infection worldwide, affecting 1 out of every 150 live-born infants worldwide (9).

Several specific bioactive and immunomodulatory factors play a role in the milk-mediated defense system against viral infections, including milk proteins, as lactoferrin, lactadherin, lactoperoxidase, lysozyme, and secretory immunoglobulins A (sIgA), but also mucins, sulfated glycolipids, glycosaminoglycans and vitamin A (6, 10–12). Despite the presence of protective factors in HM, some viruses, as human immunodeficiency virus type 1, human T-lymphotrophic virus, zikavirus, and HCMV, are transmitted from mother to infant thus the heat treatment of DM is mandatory in human milk banks (HMBs) to guarantee microbiological safety (13). The ESPGHAN Committee on Nutrition has recently advised that “future research should focus on the improvement of milk processing in HMBs, particularly of heat treatment” (14). Currently, a pasteurization process at 62.5°C for 30 min (the Holder pasteurization method, HoP) is recommended in all international guidelines for the constitution of HMBs (13, 15). However, literature indicates that HoP affects several milk components to variable degrees, with a marked effect on milk protein content and activity (16, 17). Therefore, HMBs and researchers are committed to developing novel or enhanced methods to process DM that can ensure microbial inactivation, while improving the preservation of its nutritional, immunological, and functional constituents (14). High Temperature Short Time pasteurization (HTST) was the first non-HoP technique tested to improve the nutritional and immunological quality of milk, because it has been established in the dairy industry since the 1930s. HTST in food industry is usually performed by heating thin-layered milk in continuous flow systems at 72°C for 15 seconds, although high variability on the processing equipment and conditions was recently observed for HTST when applied to HM pasteurization (18). On the basis of the existing evidence, a new small-scale continuous-flow HTST pasteurizer was recently designed and validated for treating HM by our group (19).

The present research is aimed at assessing whether and to which extent HoP and HTST have an effect on the antiviral properties of raw HM against a panel of viral pathogens causing diseases in newborns and children, as HSV-1, HSV-2, HCMV, RSV, HRhV, and HRoV.

## Materials and methods

### HM samples collection

HM samples were obtained from the HMB of the Città della Salute e della Scienza of Turin, Italy. An ethical review process was not required for this study, because it was not a clinical trial. Each milk donor involved in this research signed a written consent form, where mother's and infant's data protection was assured. Besides, donors where informed that only milk samples stored in excess to the needs of their infants should have been used for research purposes explaining the study design. Two pools of milk were obtained on two different occasions. Both pools (final volume 250 ml) included colostrum (days 1–5 postpartum), transitional milk (days 6–14 postpartum), and mature milk (beyond day 15 postpartum). Each pool contained milk samples from three donors. The donors of the first pool were different from those of the second pool. The donors cleaned their hands and breasts according to the Italian HMB guidelines (13). The milk specimens were collected in sterile bisphenol-free polypropylene bottles using a breast pump and stored by the HMB at −20°C until processed. The individual specimens were thawed overnight in refrigerated conditions, and then pooled rapidly in the morning in the HMB, and shipped refrigerated within 1 h. Upon arrival, pooled samples were processed according to the appropriate technique, and immediately separated in 10-ml aliquots, which were conveyed to the analyzing laboratory within 1 h. Raw milk was also aliquoted immediately after collection and stored as fresh and/or frozen material, as required.

### Milk pasteurization

The milk samples were processed using either HoP or HTST system. HoP was performed directly in sterile bisphenol-free polypropylene bottles using a standard HM pasteurizer (Metalarredinox, BG, Italy). HTST was performed using a patented proprietary device (European Patent n° 2974603), as previously described (19, 20). HTST-pasteurized milk was collected in sterile bisphenol-free polypropylene bottles. Four mL from each sample were skimmed by centrifugation at 2,000 g for 30 min at 4°C in sterile tubes (Eppendorf S.r.l, Milan, Italy), and then shipped refrigerated within 1 h to the laboratory for antiviral assays.

### Cells

African green monkey kidney cells (Vero) (ATCC CCL-81) and human epithelial cells Hep-2 (ATCC CCL-23) were grown as monolayers in Eagle's minimal essential medium (MEM) (Gibco/BRL) supplemented with heat-inactivated 10% fetal calf serum (FCS) (Gibco/BRL) and 1% antibiotic-antimycotic solution (Zell Shield, Minerva Biolab) at 37°C in an atmosphere of 5% of CO_2_. Human Foreskin Fibroblasts (HFF-1) (ATCC SCRC-1041) at low-passage-number (<30), African green monkey kidney epithelial cells (MA104) and human epithelial adenocarcinoma HeLa cells (ATCC CCL-2) were propagated in Dulbecco's Modified Eagle's Medium (DMEM) (Sigma-Aldrich) supplemented with FCS and antibiotic-antimycotic solution.

### Viruses

The neurovirulent strains LV (21) and MS (ATCC VR-540) of HSV-1 and HSV-2, were propagated in Vero cells at 37°C (22). HRhV 1A (ATCC VR-1559) was propagated in HeLa cells, at 33°C. HSV-1, HSV-2, and HRhV titers were determined by the standard plaque method and expressed as plaque-forming unit (PFU)/ml. A bacterial artificial chromosome (BAC)-derived HCMV strain Towne incorporating the green fluorescent protein (GFP) sequence (23) was propagated on HFF-1 and viral titres were determined by fluorescent focus assay. RSV strain A2 (ATCC VR-1540) was propagated in Hep-2 and titrated by the indirect immunoperoxidase staining procedure using an RSV monoclonal antibody (Ab35958, Abcam) (24). Human HRoV strain Wa (ATCC VR-2018) was activated with 5 μg/ml of porcine pancreatic trypsin type IX (Sigma) for 30 min at 37°C and propagated in MA104 cells by using MEM containing 0.5 μg of trypsin per ml. HCMV, RSV and HRoV titers were expressed as focus-forming units (FFU)/ml. Virus stocks were maintained frozen (-80°C).

### Viral inhibition assay

Antiviral activity of milk samples was determined by plaque reduction assays for HSV-1, HSV-2, and HRhV and by focus reduction assays for HCMV, RSV, and HRoV. Antiviral assays were performed by incubating serial dilutions of milk (from 1/1 to 1/1024 parts in medium) with virus for 1 h at 37 °C and then the mixtures were added to cells for 2 h at 37°C. After three washings with medium, monolayers were overlaid with 1.2%-methylcellulose medium with 2% FCS. The effect on HSV and HRhV infections was evaluated on pre-seeded Vero or Hela cells (10 × 10^4^) respectively, in 24-well plates infected with 200 PFU/well of HSV or 30 PFU/well of HRhV; after incubation for 24 h (HSV-2 and HRhV) or 48 h (HSV-1) cells were fixed and stained with 0.1% crystal violet in 20% ethanol and viral plaques were counted. The mean plaque count for each sample dilution was expressed as a percentage of the mean plaque count of the control (25). In HCMV inhibition assay, cells pre-seeded in 96-well plates at a density of 5.0 × 10^3^/well, were infected with 140 PFU/well of GFP-coding HCMV. After 5-day-incubation at 37°C 5% CO_2_ atmosphere, infected cells were visualized as green fibroblasts using fluorescence microscopy and counted. In RSV and HRoV inhibition assays, Hep-2 cells and MA104 were pre-seeded at a density of 1 × 10^4^/well and 1.4 × 10^4^, respectively, in 96-well plates. Cells were infected with 60 PFU/well of RSV or 200 PFU/well of HRoV and, after 16 h (HRoV) or 3 days post-infection (RSV), infected cells were fixed with cold methanol and acetone for 1 min and subjected to virus-specific immunostaining as described previously (26, 27). Fluorescent and immunostained viral foci were microscopically counted and results were reported as percentages of foci in comparison to controls. The endpoints of the assays were the effective milk dilution that reduced the viral plaque/focus formation by 50% (inhibitory dilution-50, ID50) in comparison to that in the untreated control. The ID50 of milk was calculated by using the program PRISM 4 (GraphPad Software) to fit a variable slope-sigmoidal dose–response curve. All data were generated from duplicate wells in at least three independent experiments on each HM pool.

### Cell viability assay

Cell viability was measured by the MTS [3-(4,5-dimethylthiazol-2-yl)-5-(3-carboxymethoxyphenyl)-2-(4-sulfophenyl)-2H-tetrazolium] assay. Confluent cell cultures seeded in 96-well plates were incubated with different dilutions of milk in triplicate under the same experimental conditions described for the antiviral assays. Cell viability was determined by the CellTiter 96 Proliferation Assay Kit (Promega) according to the manufacturer's instructions. Absorbances were measured using a Microplate Reader (Model 680, BIORAD) at 490 nm. Their effect on cell viability at different milk dilutions was expressed as a percentage, by comparing the absorbances of treated cells with that of the cells incubated with culture medium alone. The 50%-cytotoxic dilutions (CD50) and 95% confidence intervals (CIs) were determined with Prism 4 software.

### Statistical analysis

Statistical analysis was performed using Extra sum-of-square F test as reported in legends of figures, on GraphPad software. Significance was reported for *p* < 0.05.

## Results and discussion

### Antiviral activity of HM

**Figure 1:**
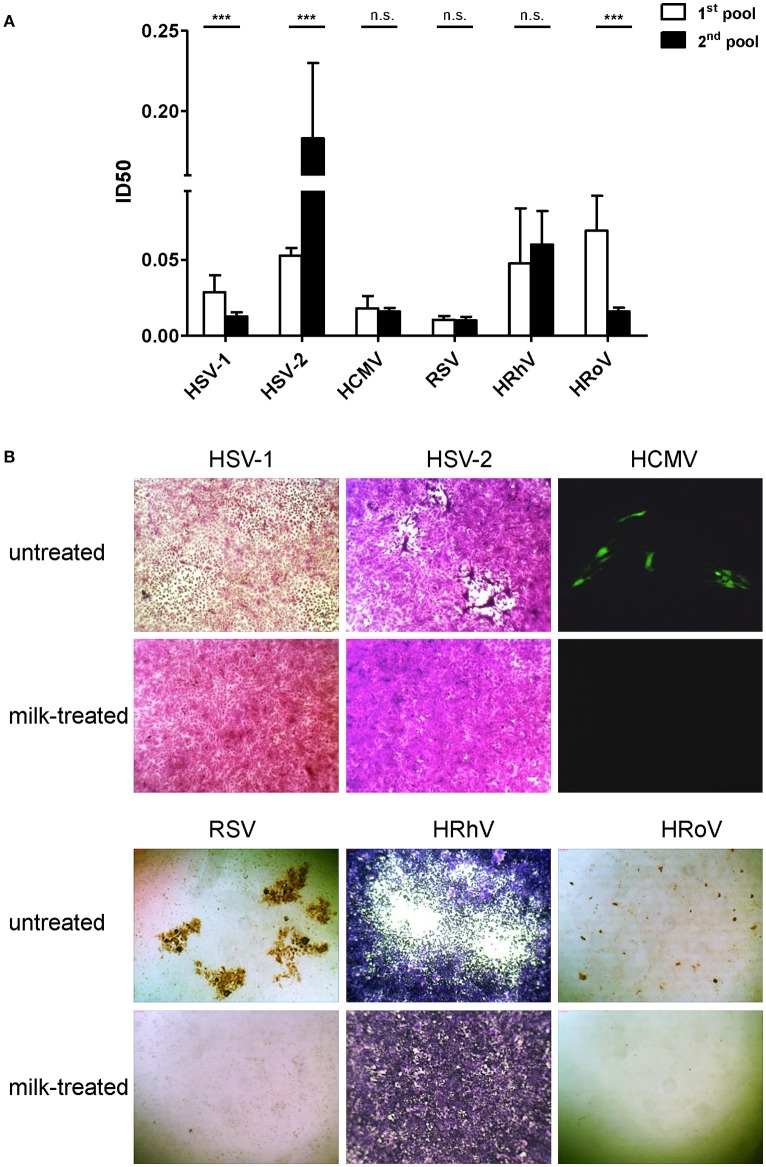
**(A)** Antiviral activities against HSV-1, HSV-2, HCMV, RSV, HRhV, and HRoV are reported as inhibitory dilution-50 (ID50) values for raw human milk pool #1 (white) and pool #2 (black bar). Data are reported as mean ID50 ± 95% confidence intervals of 4 independent experiments. ID50 values were compared using the sum-of-squares *F*-test. ^***^P < 0.001; n.s., not significant. **(B)** Representative examples of plaque reduction assays and fluorescence foci assays of antiviral assays treating infected cells with raw milk samples at a 1:2 dilution, an inhibitory dilution-100 (ID100) for all viruses. Untreated infected (upper row) and milk-treated infected (lower row) fields are reported for HSV-1, HSV-2, HCMV, RSV, HRhV, and HRoV. HSV-1, HSV-2 and HRhV plaques were visualized after crystal violet staining; RSV and HRoV foci were visualized by ICC (magnification 40X). HCMV fluorescent foci were visually counted as green cells at fluorescence microscopy (magnification 100X). HSV-1, HSV-2, and HRhV plaques are violet; HCMV infected cells are green; RSV and HRoV foci are brown.

This paper reports on the antiviral activity of raw milk and investigates the impact of two pasteurization techniques on such biological property. The first set of experiments was dedicated to assess the antiviral activity of two HM raw milk pools against a panel of viral pathogens causing diseases in newborns and children, and representing different viral structures and families: enveloped DNA viruses, as HSV-1, HSV-2, and HCMV (*Herpesviridae* family); enveloped RNA viruses, as RSV (*Paramyxoviridae* family); naked single strand RNA virus, as HRhV (*Picornaviridae* family); naked double strand RNA virus, as HRoV (*Reoviridae* family). Figure 1A reports the antiviral activity of the two HM pools, expressed as ID50, i.e., the dilution of milk sample inhibiting the 50% of infectivity. The results revealed that both pools exhibited antiviral activity against all viruses with ID50 ranging from 0.010 to 0.183. As for the viruses belonging to the *Herpesviridae* family, both pools exhibited a similar antiviral activity against HCMV, whereas a statistically significant difference in anti-HSV-1 and anti-HSV-2 activity was observed (*p* < 0.05). These results confirm previous findings that HM samples were endowed with anti-HCMV activity, although to a different extent from sample to sample and from mother to mother (6, 28). Our data evidenced a high activity against HSV-1 and HSV-2, in contrast with other studies that observed weak or no antiviral effect (29–31) for raw HM, whereas, when HM samples were aspirated from the stomachs of the infants within few hours of feeding, they were reported to reduce HSV-1 titers (31). The observed inhibitory activity of milk pools with ID50 around of 0.01 against RSV and 0.05 against HRhV supports clinical observations that maternal milk protects infants against respiratory infections, as bronchiolitis, during the first year of life, and encourages breastfeeding as an effective/inexpensive measure of prevention of lower respiratory tract infections in infancy (32, 33). However, a variable antiviral activity of HM was observed against different HRhV serotypes circulating worldwide (6). Although the anti-HRoV activity of lactoferrin and of milk fat globule membrane components that contains bioactive glycoproteins and glycolipids has been widely reported in the past, Pfaender et al. did not evidence a pronounced reduction in viral titers of HRoV by HM (30, 34). By contrast, our study reports a clear anti-HRoV effect for both milk pools, supporting protection of breastfed children against gastrointestinal viral infection. The differences in the serostatus of the donor mothers for each virus along with the interpersonal variability in the content of antiviral components of HM may explain the different extents of antiviral potencies between the two HM pools, and some inconsistencies with previous literature. Figure 1B shows representative images of the total inhibitory effect of raw milk at a 1:2 dilution against all tested viral infections. Of note, all the antiviral activities were not a consequence of cytotoxicity of HM samples, since the CD50 of milk was one or two logarithms greater than the ID50 (data not shown).

### Effect of hop and HTST methods on antiviral activity of HM

**Figure 2:**
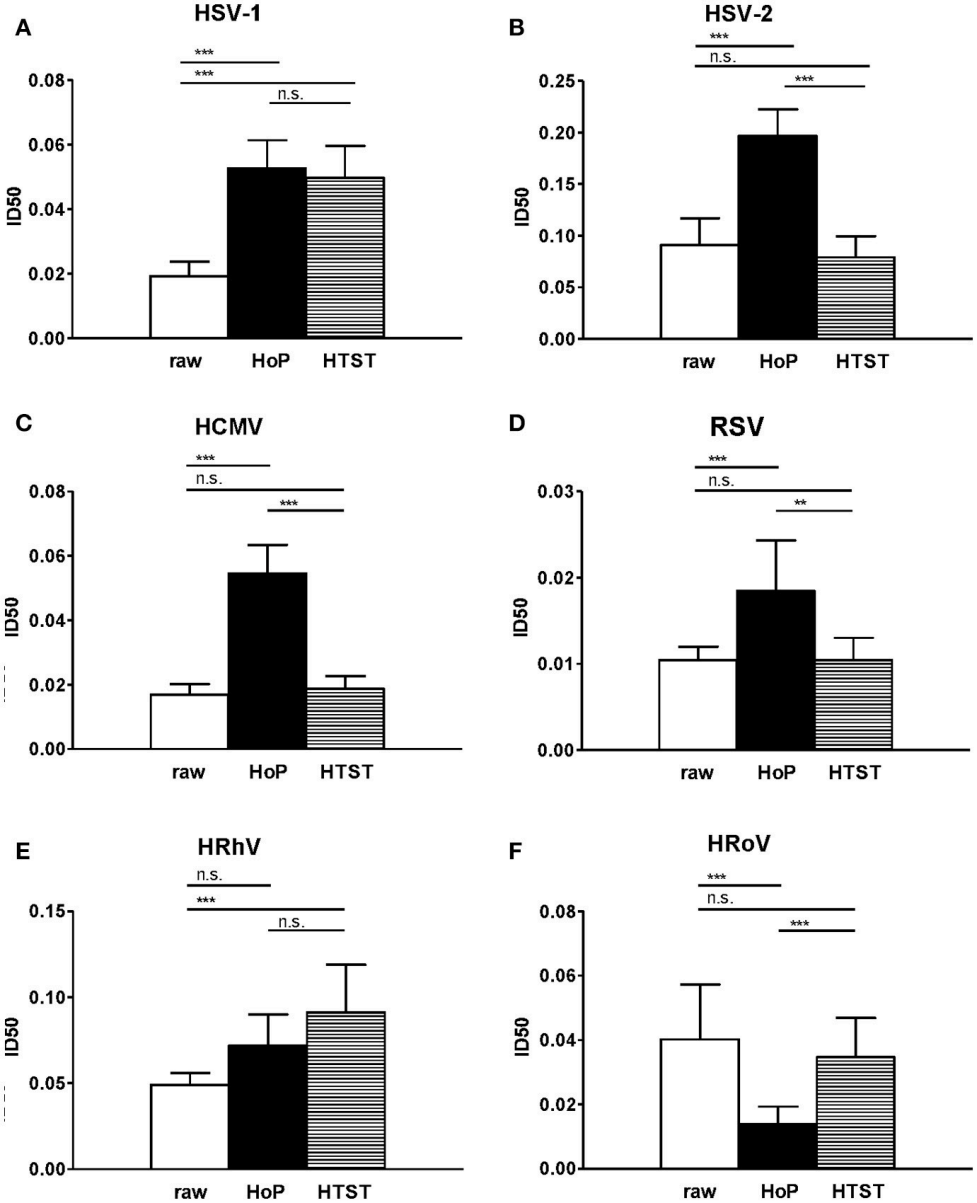
Raw milk (white), Holder pasteurized (HoP, black bar) and HTST pasteurized milk (horizontal striped bar) activities against HSV-1 **(A)**, HSV-2 **(B)**, HCMV **(C)**, RSV **(D)**, HRhV **(E)**, and HRoV **(F)** are reported as a mean ID50 ± 95% confidence intervals of two milk pools. Each pool was tested in 4 independent experiments. ID50 values were compared using the sum-of-squares *F*-test. ^**^p < 0.01; ^***^P < 0.001; n.s., not significant.

The main aim of this work was to assess the impact of two pasteurization methods, HoP and HTST, on the antiviral properties of raw HM. Therefore, in a second set of experiments, aliquots of the HM pools were pasteurized in parallel or left untreated and their antiviral activity was evaluated. Figure 2 reports the average of ID50 of the two pools, untreated or pasteurized. The results evidenced a statistically significant reduction of milk antiviral activity following HoP pasteurization against HSV-1, HSV-2, HCMV, RSV, and HRhV infections. These findings may reflect the reduction of specific components with significant immunologic and anti-infective action, such as immunoglobulins and lactoferrin, caused by HoP (16, 17). By contrast, HTST preserved the inhibitory activity of raw milk against most of the tested viruses: no significant difference was evidenced between the ID50 of raw and HTST treated HM samples against HSV-2, HCMV, RSV, and HRoV (Figure 2). These data are consistent with previous reports showing that HTST is better than HoP at preserving some biological HM properties, including the antioxidant potential, the lactoferrin content and structure, B and C vitamins, and some cytokines (18). In this experiment, the reliability of such higher biological activity retention is increased by the use of a patented equipment that has a technology readiness level (TRL) of 6, which was directly compared to a commercial HoP device normally used in HMBs. Figure 2 also showed that HoP and HTST similarly reduced the antiviral activities against HSV-1 and HRhV compared to untreated milk. Unexpectedly, HM anti-HRoV activity was increased by HoP treatment with an ID50 value of 0.014 for pasteurized milk against an ID50 value of 0.04 of raw HM. Although we do not have an explanation for this result, we can speculate that thermal treatment at 62.5°C for 30 min may alter the structure of some HM components thereby increasing their protective activity, such as the release of specific peptides active against HRoV following protein degradation.

## Conclusion

This study demonstrated that raw HM is endowed with antiviral activity *in vitro* against viral pathogens causing infections in newborns and children. Further studies are needed to investigate the clinical relevance of this activity. HoP method, currently recommended in international guidelines for HMBs, proved to significantly decrease the antiviral activity against HSV-1, HSV-2, HCMV, RSV, and HRhV but not against HRoV. By contrast, HTST preserved antiviral properties of raw HM against four out of six viruses analyzed. These data, along with previous literature, support the HTST as a valid alternative to HoP. Despite the fact that HTST provides a thermal treatment at a higher temperature than HoP (72 vs. 62.5°C, respectively), the far more rapid heating and cooling time of HTST (seconds instead of minutes, respectively) could improve the quality of the final product. This may make HTST suitable for providing the best compromise between microbiological safety and preservation of key biological components and properties of HM, including its antiviral activity.

## Author contributions

DL, LC, GM, and EB conceived and designed the study. PT and ACo, collected and pooled the HM samples. MD, MR, RF, and ACi, performed the antiviral assays. LC and MG performed the pasteurizations. MD, LC, and DL analyzed the data. DL, MD, LC, MG, PT, ACo, GM, and EB interpreted the results obtained. MD drafted the manuscript. DL, LC, GM, and EB revised the manuscript. All authors read and approved the final version of the manuscript and agreed to be accountable for all aspects of the work.

### Conflict of interest statement

LC, MG, EB, ACo have competing interests since they are the inventors of a pending patent application on the HTST pasteurizer for human milk described in the paper (Patent application no. EP 15176792.8-1358/2014). The remaining authors declare that the research was conducted in the absence of any commercial or financial relationships that could be construed as a potential conflict of interest.
